# Co-expression of MET and CD47 is a novel prognosticator for survival of luminal-type breast cancer patients

**DOI:** 10.18632/oncotarget.2385

**Published:** 2014-09-02

**Authors:** Irène Baccelli, Albrecht Stenzinger, Vanessa Vogel, Berit Maria Pfitzner, Corinna Klein, Markus Wallwiener, Martina Scharpff, Massimo Saini, Tim Holland-Letz, Hans-Peter Sinn, Andreas Schneeweiss, Carsten Denkert, Wilko Weichert, Andreas Trumpp

**Affiliations:** ^1^ Heidelberg Institute for Stem Cell Technology and Experimental Medicine (HI-STEM gGmbH), Im Neuenheimer Feld 280, 69120 Heidelberg, Germany; ^2^ Divison of Stem Cells and Cancer, Deutsches Krebsforschungszentrum (DKFZ), Im Neuenheimer Feld 280, 69120 Heidelberg, Germany; ^3^ Institute of Pathology, University Hospital Heidelberg, Im Neuenheimer Feld 224, 69120 Heidelberg, Germany; ^4^ Institute of Pathology, Charité Universitätsmedizin Berlin, Charitéplatz 1, 10117 Berlin, Germany; ^5^ National Center for Tumor Diseases (NCT), University Hospital Heidelberg, Im Neuenheimer Feld 460, 69120 Heidelberg, Germany; ^6^ Department of Biostatistics, Deutsches Krebsforschungszentrum (DKFZ), Im Neuenheimer Feld TP4, 69120 Heidelberg, Germany; ^7^ German Cancer Consortium (DKTK), 69120 Heidelberg, Germany

**Keywords:** breast cancer, prognosis, biomarker, CD47, MET, metastasis, circulating tumor cells, metastasis-stem cell

## Abstract

Although luminal-type primary breast cancer can be efficiently treated, development of metastatic disease remains a significant clinical problem. We have previously shown that luminal-type circulating tumor cells (CTCs) co-expressing the tyrosine-kinase MET and CD47, a ligand involved in cancer cell evasion from macrophage scavenging, are able to initiate metastasis in xenografts. Here, we investigated the clinical relevance of MET-CD47 co-expression in 255 hormone receptor positive breast tumors by immunohistochemistry and found a 10.3-year mean overall-survival difference between MET-CD47 double-positive and double-negative patients (p<0.001). MET-CD47 co-expression defined a novel independent prognosticator for overall-survival by multivariate analysis (Cox proportional hazards model: HR: 4.1, p<0.002) and CD47 expression alone or in combination with MET was strongly associated with lymph node metastasis. Furthermore, flow cytometric analysis of metastatic patient blood revealed consistent presence of MET^+^CD47^+^ CTCs (range 0.8 – 33.3% of CTCs) and their frequency was associated with increased metastatic spread. Finally, primary uncultured CTCs with high MET^+^CD47^+^ content showed an enhanced capacity to initiate metastasis in mice. Detection and targeting of MET and CD47 may thus provide a rational basis for risk stratification and treatment of patients with luminal-type breast cancer.

## INTRODUCTION

Breast cancer is the most frequent type of cancer in women and the second leading cause of women cancer mortality [1]. Comparison of large patient cohorts at the genomic level reveals increasing numbers of breast cancer molecular subtypes [2–4]. In clinical practice, patients are classified among four main categories using immunohistochemistry staining: luminal A, luminal B, triple negative and human epidermal growth factor 2 (HER2)-positive [5–9]. Luminal subtypes, which express the estrogen receptor (ER) and/or the progesterone receptor (PR) but not HER2, are the most frequent subtypes of breast cancer, representing more than 70% of patients [10]. Although patients with luminal-type breast cancer generally have a good prognosis (between 75% and 86% overall 5-year relative survival, [11]), many patients still succumb to the disease due to its capacity to disseminate to distant organs even decades after the removal of the primary tumor [12]. Biomarkers able to detect patients at risk to undergo metastatic spread are therefore urgently needed in order to develop early detection methods and to initiate preventive treatments for these patients, before the onset of deadly metastasis.

MET encodes a receptor tyrosine kinase, which is activated by the hepatocyte growth factor (HGF). Activation and/or overexpression of the MET oncoprotein generally was linked to poor prognosis in cancer patients, including breast carcinoma [13–17]. HGF binding to MET induces receptor dimerization and auto-phosphorylation, leading to the activation of several signaling cascades, resulting in enhanced tumor growth, survival as well as in the activation of an invasive program [18–20]. The HGF/MET axis has also been linked to the epithelial to mesenchymal transition (EMT), a cellular program, which enables differentiated epithelial cells to become more invasive and motile by partial and reversible loss of their epithelial features [21, 22]. Several inhibitors against MET are currently being evaluated in clinical trials [23–25] with very promising preliminary results in non-small cell lung cancer [26–28] and hepatocellular carcinoma [29, 30].

CD47 is the ligand of SIRPalpha, a receptor expressed by phagocytic cells such as macrophages [31]. The binding of CD47 to its receptor results in the inhibition of macrophage-mediated scavenging and has therefore been termed a “don't eat me” signal [32]. Recent studies report aberrant expression of CD47 in several cancer entities. Tumor cells expressing high levels of CD47 have the ability to escape the innate immune system and therefore survive better than CD47 negative cells in the blood of patients [33–36]. Antibody targeting of CD47 and subsequent reactivation of the innate immune system against transformed cells is showing great promises in pre-clinical studies in different hematological malignancies as well as in sarcoma [37–40].

Recently, we found that MET and CD47 are co-expressed in blood circulating metastasis-initiating cells of luminal-type breast cancers [41]. We furthermore observed that MET^+^CD47^+^ circulating tumor cells (CTCs) are mostly found among the population of CD44-expressing circulating breast cancer stem cells and that the presence of these CD44^+^MET^+^CD47^+^ “triple positive” CTCs correlates with dismal survival and increased metastasis in a small cohort of metastatic luminal breast cancer patients [41]. Based on our previous investigation, we hypothesized that MET-CD47 co-expression might be a novel prognosticator for luminal-type breast cancer patients. In order to evaluate the clinical relevance of MET-CD47 co-expression in these patients, we performed a retrospective analysis for both MET and CD47 expression in hormonal receptor positive breast cancer patients. Additionally, we measured the frequency of double positive CTCs in the blood of metastatic luminal patients by flow cytometry and evaluated its association with metastatic spread in these patients. Eventually, we compared the metastatic capacity of CTC populations isolated from two luminal breast cancer patients with different MET^+^CD47^+^ cellular contents, using our previously established CTC-xenograft assay, in order to directly evaluate the functional relevance of MET-CD47 co-expression *in vivo*.

## RESULTS

### MET expression is a prognostic marker for luminal-type breast cancer patients

**Figure 1:**
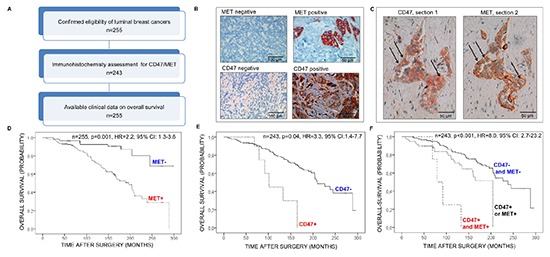
Analysis of MET and CD47 expression in luminal-type breast cancer by tissue microarray analysis **(A)** Flow-chart of the tissue microarray study. **(B)** Examples of MET (top) or CD47 (bottom) staining of luminal-type breast tumors by immunohistochemistry. **(C)** Expression of CD47 (left) or MET (right) on serial sections of a hormone receptor positive primary breast tumor. Arrows show examples of double-positive tumor cells. **(D-F)** Kaplan-Meier survival analyses of hormone receptor positive breast cancer patients based on **(E)** MET expression **(F)**, CD47 expression and **(G)** CD47-MET co- expression. Log-rank tests were used to probe for significance. Abbreviations: HR: hazard ratio; CI: confidence interval.

In order to examine the intensity and overall expression of the receptor tyrosine kinase MET in primary hormone receptor positive luminal-type mammary carcinoma, MET expression was analyzed by applying a semi-quantitative immunoreactivity scoring (IRS) system on a tissue-microarray, as described previously [42] (see clinicopathologic parameters of the study group in Table 1 and Materials and Methods for detailed methodology). As shown in Table 2, 39% (100/255) of hormone receptor positive breast tumors were scored MET positive. MET positivity was associated with a decreased mean overall-survival of 5.6 years, (p=0.001 by log-rank test, Cox proportional hazards model: hazard ratio (HR)=2.2, 95% confidence interval (CI)=1.3–3.6, p=0.002, Figure 1B and D, Table 2 and Supplementary Table 1) when compared to MET negative tumors. In addition, MET expression was found to be an independent prognostic factor for luminal-type breast cancer patients as revealed by multivariate analysis including the following co-variables: age at diagnosis, stage category, lymph node status, and grade (Cox proportional hazards model, p=0.001, HR=2.4, 95% CI=1.4–4.0, Table 3).

**Table 1:** Clinical characteristics of the cohort present on the tissue microarray

Number of patients(%)	
**Age at diagnosis**	
below mean (60.77 yrs)	115(45)
above mean (60.77 yrs)	140(55)
**Tumor stage category**	
T1	127(49.8)
T2	101(39.6)
T3	13(5.1)
T4	14(5.5)
**Lymph node status**	
N0	152(59.6)
N1	79(31.0)
N2	9(3.5)
N3	8(3.1)
missing cases	7(2.7)
**Grade**	
G1	73(28.6)
G2	144(56.5)
G3	38(14.9)
**Tumor type**	
invasive breast carcinoma (NST)	191(74.9)
lobular carcinoma	43(16.9)
other	20(7.8)
missing cases	1(0.4)

**Table 2:** Results of tissue-microarray univariate survival analyses

	Cases	Death	Overall survival (months)	Standard error	Hazard ratio (cox regression)	95% Confidence interval	P-Value (cox regression)	Log-rank Test (P-value)
**Age at diagnosis**								
below mean (60.77 yrs)	115	25	237.7	10.6				
after mean (60.77 yrs)	140	53	184.4	9.0	1.05	1.03–1.08	<0.001	0.005
**Tumor stage category**								
T1	127	36	223.5	10.0				
T2	101	33	190.8	11.7				
T3	13	2	220	34.6				
T4	14.0	7.0	154.9	29.1	1.30	1.02–1.7	0.035	0.093
Lymph node status*								
N0	152	33	232.3	8.7				
N1	79	37	174.8	13.1				
N2	9	3	163.5	29.6				
N3	8	3	59.2	6.9	2.4	1.8–3.2	<0.001	<0.001
**Grade**								
G1	73	15	241.8	12.3				
G2	144	45	198.3	10.7				
G3	38	18	153.1	15.3	2.0	1.4–2.8	<0.001	<0.001
**MET**								
negative	155	48	220.0	8.6	1.0 (reference)	1.3–3.6	**0.002**	**0.001**
**positive**	100	30	151.9	7.8	**2.2**			
**CD47**								
negative	230	70	206.1	7.9	1.0 (reference)	1.4–7.7	**0.006**	**0.004**
**positive**	13	6	117.1	14.8	**3.3**			
**MET, CD47**								
both negative	141	45	217.5	8.7	1.0 (reference)			
one positive	96	27	155.3	7.8	2.1	1.3–3.6	**0.004**	
**both positive**	6	4	93.2	14.1	**8.0**	2.7–23.2	**<0.001**	**<0.001**

* for N, 7 cases are missing

**Table 3:** Multivariate analyses of overall survival

	Hazard ratio	95%Confience interval	P-value
**Age at diagnosis**			
per year	1.043	1.02–1.07	0.001
**Tumor stage**			
T1	1.2	0.7–2.0	
T2	0.9	0.2–3.9	
T3	1.2	0.5–2.9	0.890
**Lymph node status**			
N0	1		
N1	2.6	1.6–4.3	
N2	2.7	0.8–9.6	
N3	13.5	3.4–53.2	<0.001
**Grade**			
G1	1		
G2	2.5	1.4–4.7	
G3	2.7	1.3–5.6	0.009
**MET**			
negative	1		
positive	2.4	1.4–4.0	0.001
**Age at diagnosis**			
per year	1.05	1.02–1.08	<0.001
**Tumor stage**			
T1	1.2	0.7–2.0	
T2	0.8	0.2–3.7	
T3	0.9	0.3–2.3	0.896
**Lymph node status**			
N0	1		
N1	3.5	2.0–6.2	
N2	2.6	0.7–9.6	
N3	14.6	3.7–58.0	<0.001
**Grade**			
G1	1		
G2	2.7	1.4–5.1	
G3	2.6	1.2–5.8	0.006
**CD47-MET**			
both negative	1		
one positive	2.4	1.4–4.1	
both positive	4.1	1.2–13.7	0.002

### CD47 expression is a novel prognostic marker for luminal-type breast cancer patients

Expression of CD47 (using the same IRS system) was comparably rare in hormone receptor positive breast tumors, representing only 5% of cases, (13/243 patients, Table 2). Nevertheless, univariate-analysis showed that CD47 expression was strongly associated with decreased mean overall-survival (decrease of 7.4 years, p=0.04 by log-rank test, Cox proportional hazards model: HR=3.3, 95% CI=1.4–7.7, p=0.006, Figure 1B, 1E, Table 2 and Supplementary Table 1). Moreover, CD47 expression strongly associated with the presence of lymph node metastasis: 9/14 (64.3%) patients had lymph node metastases in the CD47 positive group versus 92/245 (37.6%) in the CD47 negative group (Cochran-Armitage test for trends, p=0.01).

### Co-expression of MET and CD47 is associated with poor overall survival

Serial sections revealed the frequent presence of double positive MET-CD47 tumor cells in luminal-type breast neoplasms (Figure 1C). Subsequent analysis of the combined expression data indicated that MET and CD47 were co-expressed in 2.5% of tumors (6/243 patients, Table 2). Strikingly, patients with luminal-type breast cancer co-expressing MET and CD47 displayed a 10.3 year mean overall-survival difference compared to patients expressing neither MET or CD47 identifying a new subset of breast cancer patients with an extremely poor prognosis (Figure 1F, Table 2 and Supplementary Table 1, p<0.001 by log-rank test, Cox proportional hazards model: HR=8.0, 95% CI=2.7–23.2, p<0.001). Furthermore, co-expression of MET and CD47 defined a novel independent prognostic factor for overall-survival in these patients as revealed by multivariate analysis (Cox proportional hazards model, p=0.002, HR=4.1, 95% CI=1.2–13.7, Table 3). In addition, as for CD47 alone, CD4–MET co-expression was strongly associated with lymph node metastasis (Cochran-Armitage test for trends, p=0.04): 5/7 (71.4%) patients had lymph node metastases in the MET-CD47 double positive group versus 51/143 (35.7%) in the CD47-MET double negative group. In summary, our results suggest a link between MET-CD47 co-expression and luminal-type breast cancer dissemination as well as dismal overall-survival.

### MET^+^CD47^+^ CTCs and metastatic activity *in vivo*

The presence of 5 or more circulating tumor cells (CTCs) per 7.5 ml blood is indicative of poor progression-free and poor overall-survival in metastatic breast cancer patients, regardless of the molecular subtype [43–45]. These observations suggest that CTCs might be a source for metastasis-initiating cells [46]. Indeed, we recently reported that primary CTCs isolated from luminal-type breast cancer patients are able to initiate metastasis after transplanting them into the bone marrow of immuno-compromised mice. CTCs with metastasis-initiating cell activity express CD44, MET and CD47 [41].

In order to validate that MET-CD47 co-expression is linked to dismal outcome and metastasis *in vivo*, we first re-analyzed the previously generated flow cytometry (FACS) data of luminal-type metastatic breast cancer patient CTCs for the specific presence of double positive MET^+^CD47^+^ CTCs [41]. In all cases analyzed, CTC-positive patients (as detected by CellSearch [44] or by FACS [41]) also displayed MET^+^CD47^+^ CTCs in their blood, ranging between 0.8 – 33.3% of total detected CTCs. This corresponds to a range of 3 to 2108 MET^+^CD47^+^ CTCs per 7.5mL blood (Table 4 and Supplementary Table 2).

**Table 4:** Number and frequency of double-positive met^+^cd47^+^ CTCs in luminal-type metastatic breast cancer patients: CTCs are defined as PI-CD45-EPCAM+ cells by flow cytometry. Abbreviation: CTC: circulating tumor cell

Patient ID	Sample ID	Total volume of blood (ml)	Total number of CTCs	Number of CTCs/7.5ml blood	Total number of MET+CD47+ ctcs	Number of MET+CD47+ CTCs/7.5ml blood	Percentage of MET+CD47+ CTCs
#1	#1	7.5	6330	6330	**2108**	**2108**	**33.3%**
#3	#3	68	598	66	**178**	**20**	**29.8%**
#4	#4^a^	40	1427	268	**65**	**12**	**4.6%**
	#4^b^	7.5	289	289	**21**	**21**	**7.3%**
#5	#5	15	108	54	**14**	**0**	**13%**
#6	#6^a^	15	105	53	**10**	**5**	**9.5%**
	#6^b^	40	102	19	**19**	**4**	**18.6%**
#8	#8	72	1203	125	**133**	**0**	**11%**
#9	#9^a^	75	3827	383	**30**	**3**	**0.8%**
	#9^b^	72	3234	337	**43**	**4**	**1.3%**
#10^a^	#10^a^	20	5593	2097	**667**	**250**	**11.9%**
	#10^b^	40	1538	288	**237**	**44**	**15.4%**

a Performed before or

b after disease progression.

**Figure 2:**
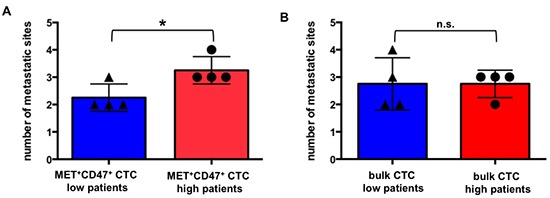
Association between MET^+^CD47^+^ CTCs and metastatic spread Association between flow cytometry-determined **(A)** MET^+^CD47^+^ CTCs (n = 8, p = 0.03, unpaired T-test)) or **(B)** bulk CTCs (n = 8, p > 0.99, unpaired T-test) and number of metastatic sites in patients. Low and high groups of patients were defined according to the median (13 for MET^+^CD47^+^ CTCs and 196 for bulk CTCs, see supplementary Table 2). Each dot represents data for one patient. CTCs are defined here as PI^-^CD45^-^EPCAM^+^ cells by flow cytometry. Data for MET^+^CD47^+^ CTCs and bulk CTCs are calculated based on datasets reported in [41]. Abbreviations: CTC: circulating tumor cell.

Second, we analyzed the impact of MET^+^CD47^+^ CTC levels in the blood of patients on metastasis development. Patients undergoing disease progression who could be additionally sampled displayed a systematic increase in their percentage of MET^+^CD47^+^ CTCs (Table 4; compare “a” before with “b” after progression). In addition, patients with high levels of MET^+^CD47^+^ CTCs (above median = 13 MET^+^CD47^+^ CTCs per 7.5mL of blood, Supplementary Table 2) had significantly more metastatic sites affected than patients with low levels of MET^+^CD47^+^ CTCs (n = 8, p = 0.03, unpaired T-test, Figure 2A). Interestingly, such an association was not observed when considering total number of FACS-detected (n = 8, p > 0.99, unpaired T-test, Figure 2B) or CellSearch-detected CTCs [41].

**Figure 3:**
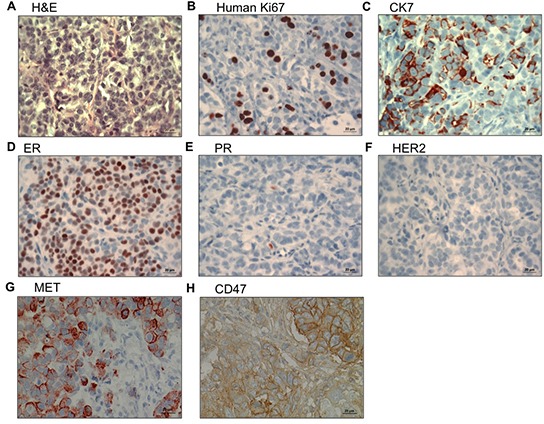
Analysis of CTC-induced bone metastasis by immunohistopathology (**A-H**) Expression of human Ki67, CK7, ER, PR, HER2, MET and CD47 in bone metastasis induced by CTCs derived from patient 8 (see Table 5), as inidicated. Abbreviations: CK7: cytokeratin 7; ER: estrogen receptor; PR: progesterone receptor.

Third, we compared the functional metastatic capacity of primary uncultured CTCs from two of these luminal-type metastatic breast cancer patients by transplanting them into the bone marrow of NOD/SCID/IL2rγc^−/−^ (NSG) mice as previously described [41]. Blood of patient 4 (47 years) was drawn one month after initial diagnosis of metastatic disease (cT2(m), cN+, G2), affecting the bones and the liver. At the time of CTC sampling, the patient had not undergone any treatment. Blood of patient 8 (44 years) was drawn nine years after initial diagnose (cT2, cN0, M0, G3) and four years after detection of metastatic disease (affecting the lymph nodes, the bones and the lungs). At the time of CTC sampling, patient 8 was not undergoing any treatment. 266 CTCs (patient 4, two mouse recipients) and 245 CTCs (patient 8, one mouse recipient) were injected into the bone marrow of NSG mice, as previously described [41]. Phenotypic analysis of the CTC populations isolated from these two patients by flow cytometry revealed the presence of 4.5% (patient 4) and 11.1% (patient 8) double-positive MET^+^CD47^+^ CTCs respectively (Table 5, see gating strategy in Supplementary Figure 1). Although comparable amounts of CTCs were injected into the three different recipient mice, only the CTCs isolated from patient 8, which contained more than twice as many MET^+^CD47^+^ cells induced the outgrowth of metastasis (Table 5 and Figure 3). The presence of functionally defined metastasis-initiating cells in the blood of patient 8 also correlated with the shorter survival of this patient after sampling compared to patient 4 (8 months vs. 22 months, Table 5).

**Table 5:** Results of circulating tumor cell xenograft assays

Patient ID	Age at time of sampling	ctcs/7.5ml blood	Number of ctcs injected/mouse	Number of MET+CD47+ CTCs injected/mouse	Number of successful grafts	Patient survival (months) after sampling
4	47	268	266	12	0/2	22
8	44	125	245	27	1/1	8

### CTC-induced metastases express MET and CD47

The bone metastasis induced by the outgrowth of the CTCs isolated from patient 8 was confirmed to be of human and epithelial origin by the detection of human Ki67 antigen and CK7 expression respectively (Figure 3). In addition, the original luminal phenotype of the primary tumor (ER positive, PR low/negative and HER2 negative) was maintained (Figure 3). Last but not least, the CTC-induced metastasis expressed MET and CD47 at high levels (Figure 3), consistent with the hypothesis that cells expressing these two molecules have an enhanced capacity to engraft *in vivo*. In summary, our results suggest a link between functional metastatic capacity and the presence of MET^+^CD47^+^ CTCs in the blood of patients.

## DISCUSSION

This study shows for the first time that CD47 expression is a prognostic marker for luminal-type breast cancer patients. Although tumors without clinical signs of metastasis only very rarely express CD47, patients expressing CD47 show a strikingly poor overall-survival as well as a higher incidence of lymph node metastasis. CD47 expression has previously been reported to prevent tumor cell phagocytosis by the cells of the innate immune system. This mechanism apparently occurs not only in leukemias [32, 35–38], but possibly also in solid tumors [33, 34, 40], in particular when tumor cells enter the systemic circulation and become CTCs. Indeed, while CD47 is only rarely expressed in non-metastatic tumors, we find it highly expressed on at least some CTCs of all breast cancer patients analyzed as well as in metastatic tumors and in all human CTC-induced metastases in xenografts (this study and [41]). These data suggest that CD47 expression is induced at the onset of luminal-type breast cancer metastasis.

Our study also confirms that MET expression is a strong prognostic indicator not only in mammary carcinoma in general [13, 15–17], but also in hormone receptor positive patients in particular [14]. HGF-MET signaling has been described as one of the inducers of the epithelial to mesenchymal transition (EMT), which leads to the reversible acquisition of an invasive and motile phenotype in carcinoma cells [21, 22, 47, 48]. Accordingly, high level of HGF in the plasma has been reported as an independent prognostic indicator of overall-survival and is associated with venous dissemination in breast cancer [49]. Luminal-type breast cancer tumors are usually rather differentiated neoplasms, which retain a more classical epithelial phenotype, as opposed to other subtypes such as triple negative tumors which are rather undifferentiated and aggressive neoplasms [4]. Up-regulation of MET signaling may therefore be a particularly critical step for the initiation of the metastatic process (involving invasion and motility throughout the systemic circulation) in the luminal breast cancer subtype.

Our study also shows for the first time that co-expression of MET and CD47 in luminal-type primary tumors represents an even better independent predictor for reduced overall-survival compared to expression of MET or CD47 alone and that it strongly correlates with lymph node metastasis. These results may therefore open new perspectives for metastasis-risk assessment of luminal-type breast cancer patients at M0 stage by simple immunohistochemical analysis of MET and CD47 expression on paraffin embedded primary tumor specimen.

Furthermore, at the metastatic stage, MET^+^CD47^+^ CTC populations were not only consistently detected in luminal-type patient blood but also associated with greater metastatic spread in patients. We previously screened metastatic patient CTC populations for their metastatic capacity and observed that CTCs only engrafted if more than 1000 cells were injected in recipient mice [41]. Here we show that only 245 CTCs isolated from a patient with a high MET^+^CD47^+^ subpopulation was sufficient for the development of new metastasis after transplantation into mice, while the same number of CTCs from another patient harboring fewer MET^+^CD47^+^ cells was unable to transplant the disease. The metastasis-initiating capacity detected in the patient with high frequency of MET^+^CD47^+^ CTCs was also associated with shorter survival (8 months after blood sampling compared to 22 months for the patient with lower frequency of MET^+^CD47^+^ CTCs). These types of *in vivo* experiments always suffer from the low number of CTCs, which can be isolated directly from the patient blood, preventing the transplantation and statistical analysis of a significant number of recipients. Nevertheless, xenografts are currently the only *in vivo* assay to functionally determine the metastatic activity of primary CTCs and have recently also been established for small cell lung cancer [50]. Importantly, all CTC-induced metastases in mice we could derive from the four different luminal patients (one in this study and three in [41]) expressed high levels of MET and CD47. In summary, our data provide correlative and functional evidence that MET^+^CD47^+^ CTCs may contain metastasis-stem cells [46].

We hypothesize that MET and CD47 co-expression provides complementary assets to luminal-type breast cancer cells during the metastatic process such as invasiveness, motility and escape from macrophage-mediated phagocytosis [51]. These features likely increase the fitness of disseminating tumor cells for metastasis initiation; therefore targeting of MET and/or CD47 signaling may provide a rational basis for novel anti-metastatic therapies. For instance, several MET inhibitors are already showing encouraging results in cancers such as hepatocellular carcinoma and non-small cell lung cancer [26–30]. In breast cancer, a pre-clinical study indicates that the MET inhibitor ARQ-197 can reduce bone metastasis induced by bone-seeking MDA-MB-231 metastatic breast cancer cells injected into mice [52]. In addition, blocking antibodies against CD47 are currently being tested in apes (lead antibody for clinics: Hu5F9-G4), and likely will soon enter phase I clinical trials [39]. As a complementary approach, anti SIRPalpha antibody targeting also showed significant efficacy in a pre-clinical study of acute myeloid leukemia [53]. Calreticulin (CRT) mediates an antagonistic signaling with respect to CD47-SIRPalpha: upon binding of CRT to its receptor, the low-density lipoprotein-related protein (LRP), on macrophages, CRT sends an “eat me” message to the innate immune system, promoting phagocytosis of SIRPalpha positive cells [54]. Antibody-mediated targeting of the CD47-SIRPalpha axis did not lead to any deleterious effect on healthy cells in mice, despite the broad expression of both molecules in normal tissues. This is probably due to the fact that normal cells do not express CRT, in striking contrast to most tumor cells [55, 56]. It is therefore expected that CD47-SIRPalpha targeting will show only modest side effects in clinical settings.

It should be noted that this study has some limitations: first, our functional *in vivo* validation should be evaluated in larger cohorts of xenografted mice in the future. Second, the specific role of CD47 and MET in the metastatic process itself still needs to be evaluated in follow-up mechanistic investigations and should be linked to molecular pathways differentially active in double-positive and double-negative CTCs. Third, although we observe a correlation between the number of MET^+^CD47^+^ CTCs and the capacity to induce new metastasis in xenografts, different combinations of mutations present in the patient tumors may also influence this activity. Fourth, this retrospective study is unable to directly link MET and CD47 co-expression in primary tumors to the occurrence of hematogenous metastasis in luminal-type breast cancer patients (only to lymph node metastasis) due to the fact that initially only patients without distant metastases were included in our cohort and that we were unable to systematically obtain detailed information on the subsequent development of metastasis in these patients. However, death of breast cancer patients almost exclusively occurs through the formation of distant metastases, and non-tumor related death usually occurs in a balanced way between groups of patients, therefore survival parameters in this setting might still tightly mirror the incidence and time kinetics of distant metastases formation. Nevertheless, the results of this study will need to be validated in independent clinical cohorts.

In conclusion, we have previously shown that circulating metastasis-initiating cells isolated from luminal breast cancer patients co-express the receptor tyrosine kinase MET as well as CD47, the ligand of SIRPalpha. However, no large-scale study had examined the clinical impact of MET and CD47 co-expression in luminal-type breast cancer patients. Here, we tested the clinical relevance of MET-CD47 co-expression in 255 luminal breast tumors and we found a strong association with dismal prognosis and metastasis development. Although this needs to be confirmed in follow-up studies, our data indicate that in the clinical setting, immunohistochemical analysis might suffice to identify double-positive luminal breast cancer patients with exceptionally dismal outcome. These patients may benefit from novel MET targeting strategies already available in clinical trials for hepatocellular carcinoma and non-small cell lung carcinoma, as well as from future CD47/SIRPalpha targeting strategies that have proven efficacy in pre-clinical trials.

## METHODS

### Study population

The use of tumor tissue for retrospective biomarker analysis was approved by the Charité ethics committee (project number EA1/139/05, date 07/28/2008). Informed consent from patients for use of biomaterials for research was obtained as part of the institutional treatment contract that was implemented in 2005. For samples collected before this implementation the need for informed consent was waived by the institutional review board of the Charité (EA1/139/05; Amendment 2013). The cohort consisted of 255 formalin-fixed paraffin embedded (FFPE) tissue samples of hormone receptor positive/Her2 negative primary invasive breast cancer. The cohort was partly established by the European FP7 METAcancer consortium (grant #200327). Corresponding clinicopathologic data were extracted from medical records and pathology reports. The majority of patients were diagnosed and treated at the Charité – University Medicine of Berlin (192/255 patients, 75.7%), the remaining patients were treated at the University teaching hospital DRK Hospitals Berlin, Köpenick (62/255 patients, 24.3%). Tumors were selected based on the availability of tissue. 243 tumor spots could be evaluated for CD47 staining, some cases could not be evaluated due to detachment of tumor tissue during the staining procedure. The follow-up data on overall survival was available for all patients and was defined as the time between first diagnosis and date of death. The mean age at diagnosis was 60.77 years (range 30–86 years). Mean follow-up time of patients still alive (overall-survival) at the endpoint of analysis was 133.4 months. 96 patients died during the follow-up period. 191 patients (74.9%) had invasive breast carcinoma of no special type (NST, ductal carcinoma), 43 had lobular carcinoma (16.9%). Few patients (20 (7.8%)) had a carcinoma of other histological type. All included patients underwent surgery between 1985 and 2008. All tumors were resected completely; none of the patients had distant metastasis or other malignant diseases at time of diagnosis, this was an exclusion criterion. Data on adjuvant therapy were available for 47.1% of the patients. For this study, hormone receptor positive status was defined as at least 10% positive tumor cells for either estrogen or progesterone receptor. All cases were Her2 negative (immunohistochemical score: 0 or 1). Cases were graded according to the Bloom–Richardson Grading modified by Elston and Ellis [57]. The distribution of clinicopathologic parameters in the study group is given in Table 1 and the flow of samples through the study is shown in the consort diagram (Figure 1A).

This retrospective study complied with reporting recommendations for tumour marker prognostic studies (REMARK) criteria [58].

### TMA generation and Immunohistochemical staining

Tissue microarrays were generated by use of a TMA precision instrument (Beecher Instruments, Silver Spring, MD, USA). Two tissue cylinders of 1 mm diameter were punched from each representative tumor-bearing donor block and transferred to the recipient block. For immunohistochemistry, the following antibodies were used: CK7 (DAKO, Glostrup, Denmark, clone OV-TL 12/30, concentration 247mg/L), human Ki67 (DAKO, clone Ki67, concentration 35mg/L), estrogen receptor (Thermo Fisher Scientific Inc, Kalamazoo, MI, USA, clone SP1, concentration 1:50), progesterone receptor (DAKO, clone PgR 636, concentration 53.8mg/L), HER2 (DAKO, polyclonal rabbit, 320mg/L), MET (Santa Cruz Technologies, Santa Cruz, CA, USA, clone C28, concentration 0.2mg/mL) and CD47 (R&D, sheep polyclonal, concentration 0.2mg/mL). For the detection of bound primary antibody, a DAKO Real Detection Multilink System with goat anti-mouse, anti-rabbit and anti-sheep antibodies were used, respectively.

As previously reported [41], after antigen retrieval (citrate buffer, pH 6.0 in a steam pot) sections were blocked for endogenous Avidin/Biotin activity (Linaris, Dossenheim, Germany). Sections were then incubated for 30 minutes at room temperature with the primary antibody, washed and subsequently incubated with the respective secondary antibody for 20 minutes at room temperature. Sections were then incubated with horseradish peroxidase (HRP) for 20 minutes at room temperature. Sections were counterstained with hematoxylin. Isotype-matched mouse monoclonal antibodies were used as negative controls.

### Histopathological examination

Expression of MET and CD47 was scored by two experienced histopathologists (ASt and WW) using a multi-headed microscope. The evaluating pathologists were blinded to clinical data. Unclear cases were discussed until consensus was achieved. Staining of MET and CD47 in tumor tissue was scored by applying a semi-quantitative immunoreactivity scoring (IRS) system, as described previously [42]. Briefly, category A documented the intensity of staining as 0 (no immunostaining), 1 (weak), 2 (moderate) and 3 (strong). Category B documented the percentage of immunoreactive cells as 0 (none), 1 (1–10%), 2 (11–50%), 3 (51–80%) and 4 (>80%). Multiplication of categories A and B resulted in an IRS ranging from 0 to 12 for each individual case. A case was scored as positive when tumor cells expressed high levels of protein (scoring between 7 and 12) while the cases scored negative corresponded to tumors showing low levels of protein expression (scoring between 0 and 6).

### Statistical analyses

Correlation of patterns with clinicopathologic data including nodal status was done by Cochran-Armitage test for trends. Overall-survival was estimated using the Kaplan-Meier method, with a log-rank test to probe for significance. Hazard ratios were calculated and multivariate survival analyses were done by the Cox proportional hazards model. Statistical analyses of tissue-microarrays were performed using SPSS Statistics 20 (IBM, Ehningen, Germany). Statistical analysis of results presented in Figure 2 was carried out on version 6.0 of GraphPad Prism (La Jolla, CA, USA), using an unpaired T-test to probe for significance. P-values <0.05 were considered significant. All statistical tests were used in the two-sided variant.

### Patient samples for CTC collection

Blood samples were collected in tubes containing EDTA after informed consent of luminal metastatic breast cancer patients according to the outlines of the studies S-295/2009, approved by the Heidelberg Medical Faculty Ethics Commission.

### Flow-cytometric analyses of CTCs

As described in [41], patient CTCs were characterized by multi-parameter flow-cytometry (CYAN, DAKO, Eching, Germany and LSRII, BD Biosciences) after depletion of hematopoietic cells using the RosetteSep^®^ kit (StemCell Technologies, kit number 15167). The antibodies used for these experiments are the following: CD45-PB (clone HI30, Biolegend, San Diego, California, USA), EPCAM-FITC (clone HEA-125, Miltenyi, Bergisch Gladbach, Germany), CD47-PE (clone B6H12, BD Biosciences) and MET-APC (clone 95106, R&D, Minneapolis, Minnesota, USA). Propidium Iodide (Sigma-Aldrich, St. Louis, Missouri, USA) was used to exclude dead cells. FACS analyses were carried out using version 9.5.2 of the Flowjo software (Ashland, OR, USA).

### Metastatic xenograft model

Mice were treated according to the German authorization numbers G-114/08 and G-240/11, as previously reported [41]. Briefly, 2-month-old NOD/SCID/IL2rγc^−/−^ (NSG) female mice were anesthetized by intra-peritoneal injection of 10μL per g of a solution of 4,5 mg/kg Xylazinhydrochlorid and 90 mg/kg Ketamin. RosetteSep^®^-enriched CTCs were injected in the femurs of anesthetized mice, in a 20μL solution of 1:4 10mg/mL Matrigel (BD Biosciences, Heidelberg, Germany) in phosphate buffered saline (PBS, SIGMA, St Louis, MO, USA). During narcosis, 90-day release 0.18mg pellets of Estradiol (Innovative Research of America, Sarasota, FL, USA) were implanted subcutaneously and renewed every 90 days during gaseous narcosis. Mice were monitored for up to 12 months after injection.

## SUPPLEMENTARY FIGURE AND TABLES


