# Supplementary material for: Co-expression of MET and CD47 is a novel prognosticator for survival of luminal-type breast cancer patients
Source: Oncotarget. 2014 Sep 2;5(18):8147–60. doi: 10.18632/oncotarget.2385 (PMC4226673; doi:10.18632/oncotarget.2385)
Supplement: Supplementary file 1 [file oncotarget-05-8147-s001.pdf]

SUPPLEMENTARY FIGURE AND TABLES

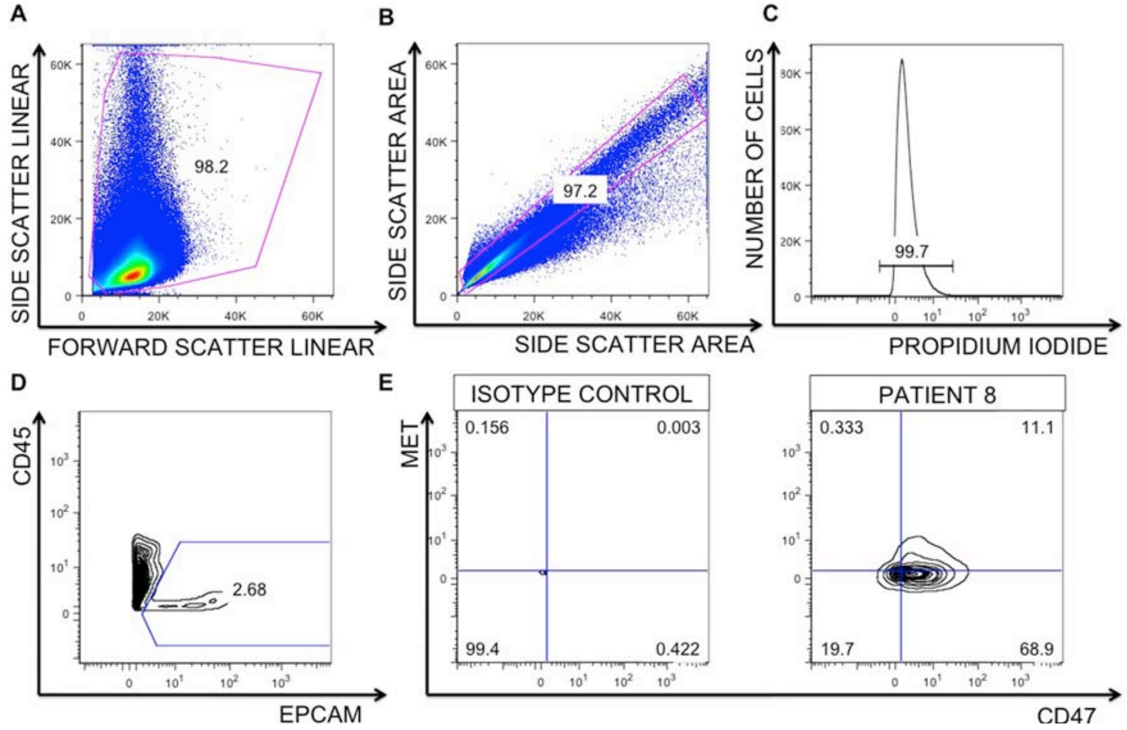

Supplementary Figure S1: Example of patient CTC profiling by flow cytometry for CD47 and MET co-expression. **(A)** Gating strategy to select cells **(B)** as singlets, and **(C)** as living cells (propidium idodide negative). **(D)** Gating strategy to select EPCAM^{+}CD45^{-} CTCs. **(E)** Staining of CD47 and MET using an isotype control (left) or using specific antibodies (right) on CTCs from patient 8. Abbreviations: CTC: circulating tumor cell (defined here as PI^{-}CD45^{-}EPCAM^{+} cells).

Supplementary Table S1. Number of patients at risk (in reference to 1D, E and F)

Months	MET^-	MET^+	$CD47^-$	$CD47^+$	MET^-CD47^-	MET^+ or $CD47^+$	MET^+ and $CD47^+$
0	56	199	230	13	141	96	6
6	55	198	230	13	140	95	5
12	55	196	227	13	140	93	5
24	55	193	224	13	140	91	5
36	55	187	219	13	138	88	5
48	53	177	208	13	133	82	5
60	50	142	173	12	116	62	5
72	48	115	148	11	109	44	3
84	47	107	143	5	105	40	2
96	46	104	139	4	102	40	1
108	46	98	134	3	98	38	1
120	46	90	126	3	94	33	1
132	46	86	123	3	92	32	1
144	46	79	117	2	88	30	0
156	42	71	106	1	84	23	0
168	30	44	69	0	62	6	0
180	26	31	54	0	53	1	0
192	21	25	43	0	42	1	0
204	12	15	24	0	24	0	0
216	8	11	17	0	17	0	0
228	7	9	14	0	15	0	0
240	7	6	10	0	11	0	0
252	6	6	10	0	10	0	0
264	6	6	10	0	10	0	0
276	2	1	2	0	2	0	0
288	2	1	1	0	1	0	0
300	0	0	0	0	0	0	0

Supplementary Table S2. Numbers of metastatic sites as well as bulk and MET^{+}CD47^{+} CTCs of hormonal receptor positive breast cancer patients as determined by flow cytometry. CTCs are defined here as PI^{-}CD45^{-}EPCAM^{+} cells by flow cytometry. Data are calculated based on the analysis reported in [41] and are the basis for Figure 2.

Patient #	Age at diagnose of metastasis	Age at CTC screening	Number of metastatic sites	CTCs/7.5mL blood by FACS (PI^{-}CD45^{-} EPCAM^{+})	MET^{+}CD47^{+} CTCs/7.5mL blood (FACS)
1	58	59	3	6330	2108
3	58	62	3	66	20
4[a]	47	48	2	268	12
5	38	41	2	54	7
6[a]	34	34	2	53	5
8	41	44	4	125	14
9[a]	48	52	3	383	3
10[a]	66	69	3	2097	250
	**Age at diagnose of metastasis**	**Age at CTC screening**	**Number of metastatic sites**	**CTCs/7.5mL blood by FACS (PI^{-}CD45^{-} EPCAM^{+})**	**MET^{+}CD47^{+} CTCs/7.5mL blood (FACS)**
**MEDIAN**	48	50	3	196	13
